# Designing of Carbon Nitride Supported ZnCo_2_O_4_ Hybrid Electrode for High-Performance Energy Storage Applications

**DOI:** 10.1038/s41598-020-58925-4

**Published:** 2020-02-06

**Authors:** Meenu Sharma, Anurag Gaur

**Affiliations:** Department of Physics, National Institute of Technology, Kurukshetra, 136119 Haryana India

**Keywords:** Energy science and technology, Nanoscience and technology

## Abstract

This study reports a unique graphitic-C_3_N_4_ supported ZnCo_2_O_4_ composite, synthesized through a facile hydrothermal method to enhance the electrochemical performance of the electrode. The g-C_3_N_4_@ZnCo_2_O_4_ hybrid composite based electrode exhibits a significant increase in specific surface area and maximum specific capacity of 157 mAhg^−1^ at 4 Ag^−1^. Moreover, g-C_3_N_4_@ZnCo_2_O_4_ electrode maintained significant capacity retention of 90% up to 2500 cycles. Utilizing this composite in the development of the symmetric device, g-C_3_N_4_@ZnCo_2_O_4_//g-C_3_N_4_@ZnCo_2_O_4_ displays a specific capacity of 121 mAhg^−1^. The device exhibits an energy density of 39 Whkg^−1^ with an equivalent power density of 1478 Wkg^−1^. A good cycling stability performance with an energy efficiency of 75% and capacity retention of 71% was observed up to 10,000 cycles. The superior performance of g-C_3_N_4_@ZnCo_2_O_4_ is attributed to the support of the g-C_3_N_4_ which increases the surface area, electroactive sites and provides chemical stability for electrochemical performance. The outstanding performance of this exclusive device symbolizes remarkable progress in the direction of high-performance energy storage applications.

## Introduction

The need for advanced energy storage devices is in great demand because of numerous portable electronic devices and the emergence of hybrid electric vehicles in modern society. However, most of these new inventions require high-performance energy storage with high energy and power densities^1,2^. Among different energy storage devices, electrochemical capacitors with high power density and batteries with high energy density have gained significant research attention to meet the needs of increasing demand for energy storage applications. Several materials like metal hydroxides/oxides, activated carbon and conducting polymers have been explored as electrodes material for energy storage applications, with an emphasis on exploring new composite to boost the electrochemical efficiency of the energy storage device^3^. Lithium-ion batteries can store high energy up to 150–200 Whkg^−1^, but confined to low power density and poor cycle life, whereas supercapacitors have high power energy, low cost and low maintenance for high-power delivery applications^4^. Therefore, the fabrication of rationally designed hybrid electrode materials is in highest demand to enhance the electrochemical performance of energy storage applications.

Transition metal oxides with various nanostructures have been used widely for electrode material because of their high theoretical capacitance. In the past years, ZnCo_2_O_4_ has aroused one of the most capable electrode materials by researchers toward their applications for supercapacitors because of their high electrochemical activities and speedy faradic reactions^5^. However, it suffers relatively poor electrical conductivity and a considerable reduction in capacitance during long cycling life and low surface area^6^.

Among the various carbon-based nanostructures, graphitic carbon nitride (g-C_3_N_4_) is a soft polymer with porous nature and sheet-like crystallite has attracted considerable attention due to its highly active nitrogen sites, excellent physical and chemical strength, and low-cost feature^7^. In the application of water splitting, waste-water detoxification, solar cells and supercapacitors, g-C_3_N_4_ carbon-based materials show superior performance because of its outstanding optical properties, high mechanical strength and thermal conductivity^8^. The nitrogen presence in g-C_3_N_4_ provides more active sites and advances the capacitance while preserving the cyclability of the electrochemical device^9,10^. Therefore, the composite of g-C_3_N_4_ and ZnCo_2_O_4_ could have great advancement in rate capability and specific capacitance^9^. To date, only a few studied of g-C_3_N_4_ with Ni(OH)_2_^11,12^, MnO_2_^13^ and NiCo_2_O_4_^14^ for energy storage application have been reported.

In this work, we synthesize g-C_3_N_4_ hybridized ZnCo_2_O_4_ composite for the electrode material in order to develop a high-performance symmetric supercapattery device. This hybrid g-C_3_N_4_@ZnCo_2_O_4_ composite exhibits excellent electrochemical performance through a specific capacity of 154 mAhg^−1^ at 4 Ag^−1^ with 90% of capacity retention up to 2500 cycles. Further g-C_3_N_4_@ZnCo_2_O_4_ based solid-state symmetric supercapattery device g-C_3_N_4_@ZnCo_2_O_4_//g-C_3_N_4_@ZnCo_2_O_4_ has been assembled, which exhibits a 39 Whkg^−1^ of energy density with an equivalent power density of 1478 Wkg^−1^.

## Results and Discussion

**Figure 1:**
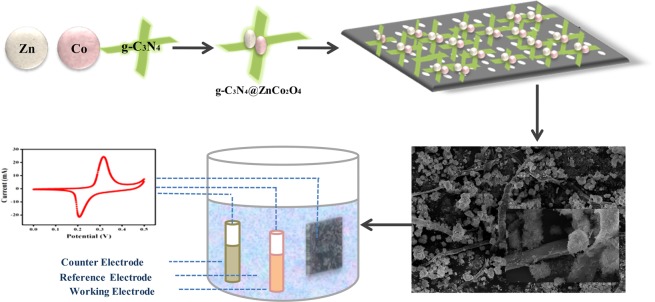
Schematic illustration of g-C_3_N_4_@ZnCo_2_O_4_ composite mechanism along with the testing of the fabricated electrode.

The synthesis of g-C_3_N_4_@ZnCo_2_O_4_ hybrid composite with high surface area is performed through a simple hydrothermal process. The schematic illustration of g-C_3_N_4_@ZnCo_2_O_4_ formation mechanism along with the testing of the fabricated electrode is shown in Fig. 1. The obtained g-C_3_N_4_@ZnCo_2_O_4_ based electrode is incorporated into a solid-state symmetric Supercapattery device, which possesses a favourable energy density and power density.

**Figure 2:**
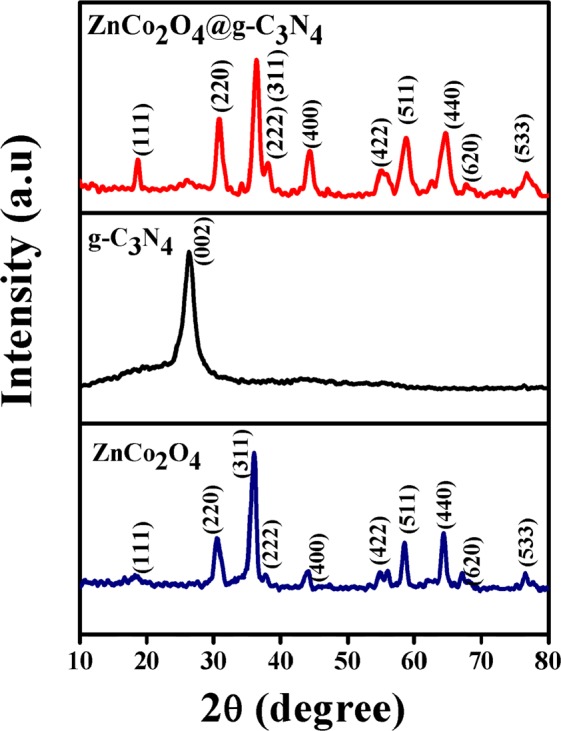
XRD patterns of g-C_3_N_4_@ZnCo_2_O_4_, ZnCo_2_O_4_ and g-C_3_N_4_.

**Figure 3:**
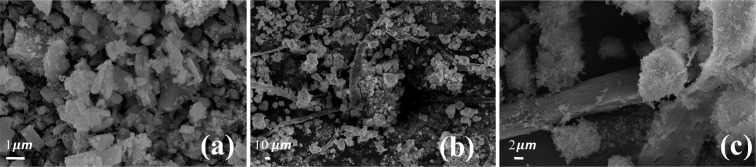
SEM images: (**a**) ZnCo_2_O_4_, (**b**,**c**) g-C_3_N_4_@ZnCo_2_O_4_ composite.

**Figure 4:**
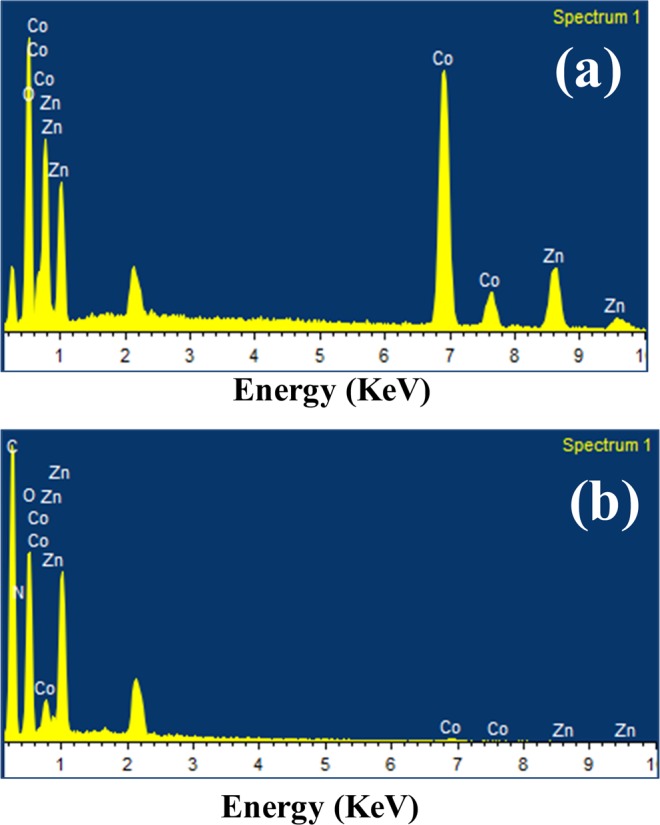
EDX of (**a**) ZnCo_2_O_4_ (**b**) g-C_3_N_4_@ZnCo_2_O_4_ composite.

XRD pattern of g-C_3_N_4_, ZnCo_2_O_4_ and g-C_3_N_4_@ZnCo_2_O_4_ is displayed in Fig. 2. For ZnCo_2_O_4_ sample, all the peaks of the XRD patterns are well matched to the (JCPDS No. 14-0117) whereas XRD pattern of g-C_3_N_4_@ZnCo_2_O_4_ shows the diffraction peaks corresponding to ZnCo_2_O_4_ and g-C_3_N_4_, implying the good formation of g-C_3_N_4_@ZnCo_2_O_4_ composites. Figure 3(a) shows the SEM micrographs of pristine ZnCo_2_O_4_ and Fig. 3(b,c) shows the SEM micrographs of g-C_3_N_4_@ZnCo_2_O_4_ composites at different magnifications. The SEM images of pristine ZnCo_2_O_4_ shows the agglomerate clusters, whereas g-C_3_N_4_@ZnCo_2_O_4_ composites show clusters with fibres distribution. The visible spaces between clusters could provide a channel for the movement of electrolyte ions and achieve a high specific surface area. Figure 4(a) represents the EDX pattern of ZnCo_2_O_4_ and Co, Zn, and O three kinds of elements are detected, whereas Fig. 4(b) represents the C, N, Co, Zn, and O five kinds of elements for composite g-C_3_N_4_@ZnCo_2_O_4_.

**Figure 5:**
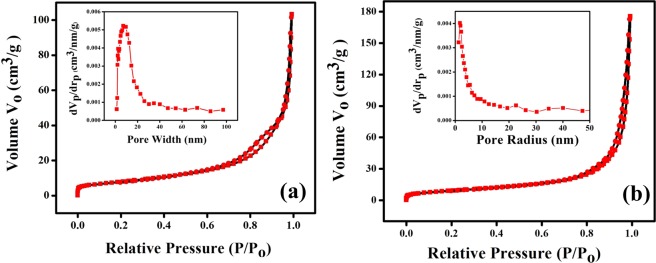
N_2_ adsorption/desorption isotherms with corresponding pore size distributions: (**a**) pure ZnCo_2_O_4_ and (**b**) g-C_3_N_4_@ZnCo_2_O_4_ composite.

The evaluation of the specific surface area and porous nature of pristine ZnCo_2_O_4_ and g-C_3_N_4_@ZnCo_2_O_4_ has been done using the N_2_ adsorption-desorption isotherms. Figure 5(a,b) displays the nitrogen adsorption-desorption measurements with inset the BJH pore size distribution plot for pristine ZnCo_2_O_4_ and g-C_3_N_4_@ZnCo_2_O_4_, respectively. The isotherms of both the samples show IV type behaviour with hysteresis loops, indicating the mesoporous nature. The Specific surface area calculated from the Brunauer-Emmett-Teller (BET) isotherms is 46 m^2^g^−1^ for a g-C_3_N_4_@ZnCo_2_O_4_ sample which significantly greater than the surface area for pristine ZnCo_2_O_4_ (22 m^2^ g^−1^). This increase in surface area of g-C_3_N_4_@ZnCo_2_O_4_ composite could be ascribed to the incorporation of Carbon nitride, which leads to the formation of more numbers of pores^15^. The BJH plot for both the samples displays the pore size within the range of 2 to 50 nm, which shows the mesoporous nature of the samples. The mesoporous nature of the sample could provide effortless access for electrolyte ions with a short diffusion path for electrochemical reactions^16,17^.

**Figure 6:**
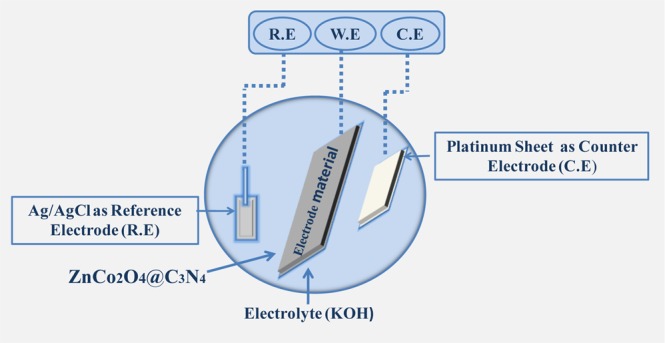
The Schematic illustration of testing a single electrode in the three-electrode electrochemical system.

**Figure 7:**
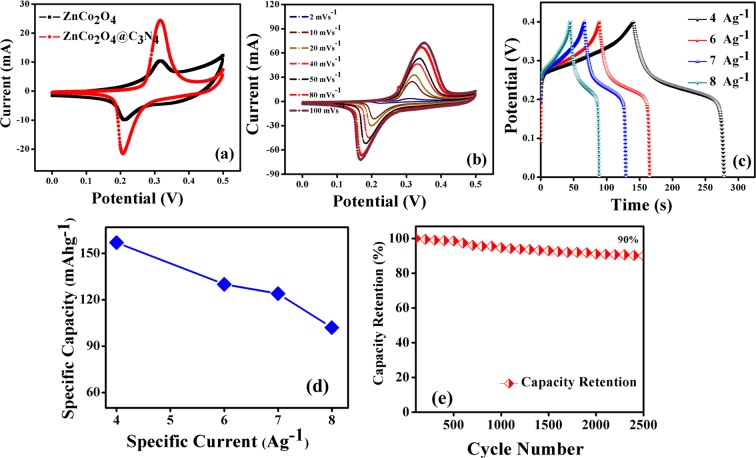
(**a**) CV curves of ZnCo_2_O_4_ and g-C_3_N_4_@ZnCo_2_O_4_ at a constant scan rate of 20 mVs^−1^. (**b**) CV curves for g-C_3_N_4_@ZnCo_2_O_4_ for different scan rates. (**c**) GCD curves for g-C_3_N_4_@ZnCo_2_O_4_ at different specific current. (**d**) Specific Capacity as a function of specific current for g-C_3_N_4_@ZnCo_2_O_4_ sample. (**e**) Capacity retention up to 2500 cycles for g-C_3_N_4_@ZnCo_2_O_4_ sample.

The electrochemical analysis of fabricated electrodes in a three-electrode configuration is schematically illustrated in Fig. 6. The CV curves of pristine ZnCo_2_O_4_ and g-C_3_N_4_@ZnCo_2_O_4_ electrodes at a constant scan rate of 20 mVs^−1^ in the potential range of 0.0 to 0.5 V is shown in Fig. 7(a). The CV curve for g-C_3_N_4_@ZnCo_2_O_4_ shows a very high amplitude of current as compared to ZnCo_2_O_4_ electrode, resulting in an enhancement in the specific Capacity^18^. A pair of redox peaks is primarily raised from faradic redox reactions linked to M-O/M-O-OH (M = Zn or Co)^19,20^. Figure 7(b) demonstrates the CV curves of g-C_3_N_4_@ZnCo_2_O_4_ at different scan rates from 2–100 mVs^−1^. The amplification and shift in the position of the anodic and cathodic peak are appeared with the increase of scan rates, indicating the quick and reversible redox responses happening at the electro/electrolyte interface^21,22^. The GCD curves of ZnCo_2_O_4_ and g-C_3_N_4_@ZnCo_2_O_4_ electrodes at a constant specific current of 4 Ag^−1^ is shown in Fig. S1. Figure 7(c) displays the GCD curves of g-C_3_N_4_@ZnCo_2_O_4_ at a various specific current of 4, 6, 7 and 8 Ag^−1^. Figure 7(d) represents the specific discharge capacity of the g-C_3_N_4_@ZnCo_2_O_4_ electrode evaluated from GCD curves. The maximum specific capacity obtained for the hybrid g-C_3_N_4_@ZnCo_2_O_4_ electrode is 157 mAhg^−1^ at 4 Ag^−1^. The superior capacity retention of 90% up to 2500 constant GCD cycles at 10 A g^−1^ is demonstrated by the g-C_3_N_4_@ZnCo_2_O_4_ based single electrode.

**Figure 8:**
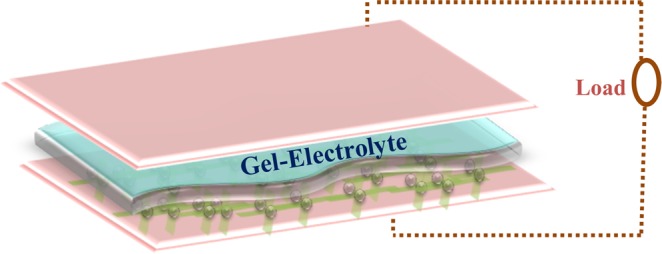
Schematic illustration of assembled g-C_3_N_4_@ZnCo_2_O_4_//g-C_3_N_4_@ZnCo_2_O_4_ symmetric supercapattery device using gel electrolyte.

**Figure 9:**
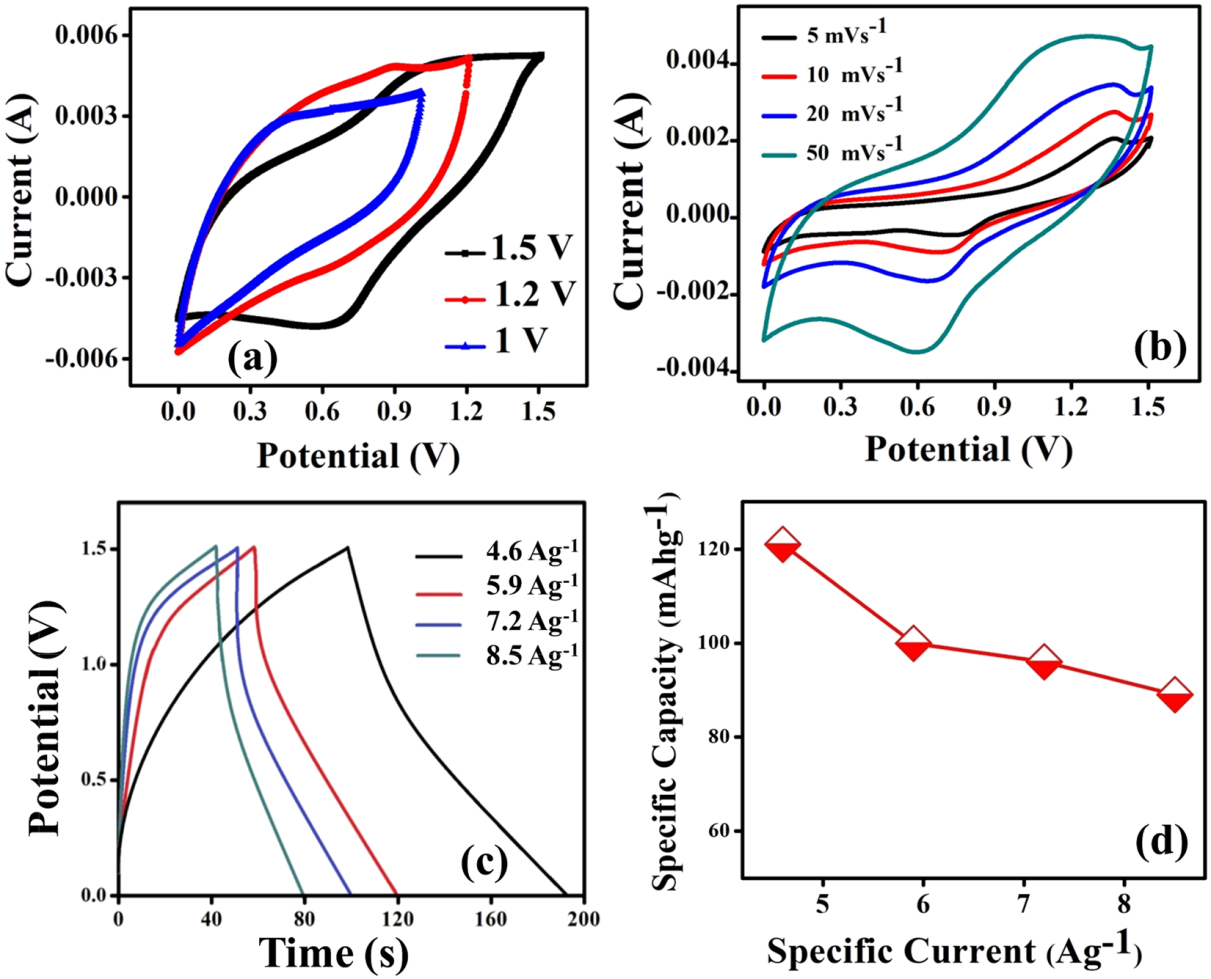
(**a**) CV curves of the g-C_3_N_4_@ZnCo_2_O_4_//gel electrolyte//g-C_3_N_4_@ZnCo_2_O_4_ symmetric supercapattery device measured at different potential windows. (**b**) CV curves of the g-C_3_N_4_@ZnCo_2_O_4_//gel electrolyte//g-C_3_N_4_@ZnCo_2_O_4_ measured at different scan rates ranging from 5 to 50 mVs^−1^ within the potential window of 0 to 1.5 V. (**c**) Galvanostatic charge-discharge curves of g-C_3_N_4_@ZnCo_2_O_4_//gel electrolyte//g-C_3_N_4_@ZnCo_2_O_4_ device at various specific current values. (**d**) Specific capacity as a function of specific current for g-C_3_N_4_@ZnCo_2_O_4_//gel electrolyte//g-C_3_N_4_@ZnCo_2_O_4_ device.

We further assembled the symmetric devices, referred to supercapattery, based on g-C_3_N_4_@ZnCo_2_O_4_ electrodes having sandwiched construction of g-C_3_N_4_@ZnCo_2_O_4_//gel electrolyte//g-C_3_N_4_@ZnCo_2_O_4_ as shown in Fig. 8. This configuration of the symmetric device with the gel electrolyte not only prevents the leakage of aqueous electrolyte but also expand the potential window^23,24^. The optimization of a suitable operating potential window is an important assignment for supercapattery device to sustain the electroactivity of working electrodes and electrolyte^25^. So, initially CV measurements were performed at the different operating potential window as shown in Fig. 9(a). The potential window from 0 to 1.5 V is found to be constant for the assembled supercapattery device which is higher than the symmetric device in liquid electrolytes^26,27^.

**Figure 10:**
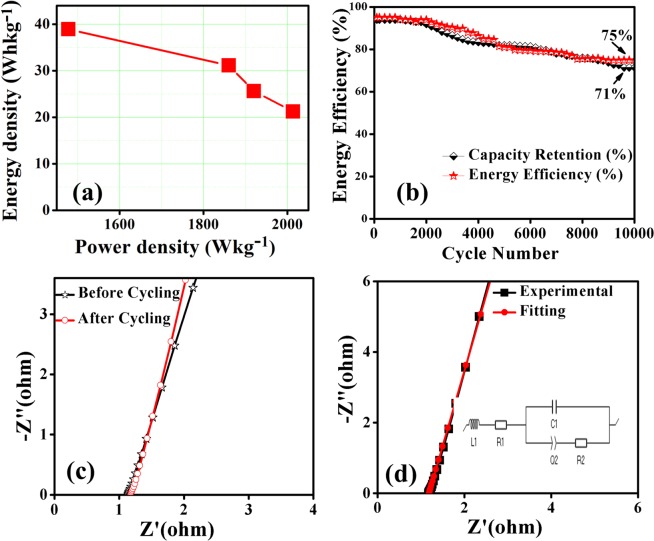
(**a**) Ragone plot for g-C_3_N_4_@ZnCo_2_O_4_//gel electrolyte//g-C_3_N_4_@ZnCo_2_O_4_ symmetric device. (**b**) Capacity retention and energy efficiency of g-C_3_N_4_@ZnCo_2_O_4_//gel electrolyte//g-C_3_N_4_@ZnCo_2_O_4_ symmetric device for 10000 cycles at 15 Ag^−1^. (**c**) Nyquist impedance plot of g-C_3_N_4_@ZnCo_2_O_4_//gel electrolyte//g-C_3_N_4_@ZnCo_2_O_4_ symmetric device before and after cycling. (**d**) Fitted Nyquist plot of g-C_3_N_4_@ZnCo_2_O_4_//gel electrolyte//g-C_3_N_4_@ZnCo_2_O_4_ symmetric device and the equivalent circuit (insert).

Figure 9(b) displays the CV curves of the electrodes between 0.0–1.5 V at a different scan rate of 5, 10, 20, and 50 mVs^−1^, respectively. The similarity in CV curves of the device with increasing scan rates indicates the excellent rate capability of the device^28,29^. The GCD measurements at a specific current of 4.6, 5.9, 7.2 and 8.5 Ag^−1^ is shown in Fig. 9(c) within the potential window of 0 to 1.5 V. By using these GCD curves, the specific discharge capacity (*Q*_s_) is calculated and plotted as a function of specific current in Fig. 9(d). The *Q*_s_ values calculated from GCD curves of the symmetric device are 121, 100, 96 and 89 mAhg^−1^ for 4.6, 5.9, 7.2 and 8.5 Ag^−1^, respectively. To estimate the overall efficiency of the assembled symmetric device, energy and power density were calculated and illustrated as a Ragone plot in Fig. 10(a). This device achieved a maximum energy density of 39 Whkg^−1^ at a power density of 1478 Wkg^−1^. The g-C_3_N_4_@ZnCo_2_O_4_//gel electrolyte//g-C_3_N_4_@ZnCo_2_O_4_ symmetric device maintained an energy efficiency of 75% in addition to a capacity retention of 71% up to 10000 continuous cycles at 15 Ag^−1^ as shown in Fig. 10(b). This demonstrates long-term electrochemical stability of the symmetric device. To further assess the electrochemical properties of device, the EIS analysis in Fig. 10(c) shows the solution resistance (R_s_) before and after 10000 cycles of GCD. In the high-frequency region, R_s_ is the intercept on the Z’-axis, which is a combination of resistance in the electrolyte and the material electrode^30^. The calculated value of R_s_ before and after 10000 cycles is estimated to be 1.1 and 1.5 Ω, respectively, representing the stability of the synthesised material for fabricated supercapattery device. Moreover, the Nyquist plot for g-C_3_N_4_@ZnCo_2_O_4_//gel electrolyte//g-C_3_N_4_@ZnCo_2_O_4_ symmetric device was fitted as shown in Fig. 10(d) with its corresponding equivalent circuit displayed in the inset.

## Experimental

### Synthesis of the g-C_3_N_4_@ZnCo_2_O_4_ composite

All the reagents were of analytical quality and used without additional refinement. The g-C_3_N_4_ was prepared through simple pyrolysis of urea under ambient atmosphere. Initially, urea was dried at 80 °C for 24 hours and it was placed in a crucible for heat treatment in an oven. After heating the precursor at 550 °C for 3 hours, g-C_3_N_4_ of yellow-coloured was obtained. Then, 1 mM of Zinc Nitrate, 2 mM of Cobalt Nitrate and 5 mM of urea was dissolved properly in 40 mL deionized (DI) water by stirred for 30 min. The prepared 10 mg of the obtained g-C_3_N_4_ was then mixed with the prepared solution and stirred firmly for an additional 1 h. Then the entire solution was moved to a 70 mL Teflon-lined autoclave box and heated to 180 °C for 12 h. After the autoclave box was cooled to room temperature, the precursor was collected, rinsed consecutively with DI wand ethanol. Afterwards, the final product was calcinated at 450 °C for 2 h.

### Electrochemical analysis of electrode and symmetric device

To fabricate the working electrodes, 80 wt% of as-synthesized g-C_3_N_4_@ZnCo_2_O_4_, 10 wt%) of carbon black and a polymer binder (polyvinylidene fluoride; PVDF, 10 wt%) was mixed using few drops of N-Methyl-2-pyrrolidone (NMP). The resulting slurry was applied on a flexible nickel mesh substrate (current collector) using the drop-casting method, followed by drying it at 120 °C for 12 h in an oven. The electrochemical measurements of electrodes were carried out on Biologic potentiostat workstation in a 6 M KOH electrolyte using cyclic voltammetry (CV), galvanostatic charge-discharge (GCD) and electrochemical impedance spectroscopy (EIS) measurements. In three electrodes configurations, the as-prepared electrode was used as a working electrode, Ag/AgCl and platinum were used as reference electrode and counter electrode, respectively.

The specific discharge capacity, *Q*_s_ (mAhg^−1^) and energy efficiency, η(%) of electrodes was calculated from galvanostatic charge-discharge measurements by using the following equation

1 $${Q}_{{\rm{S}}}=\frac{{\rm{I}}\Delta {\rm{t}}}{3.6\,{\rm{m}}}$$

2 $${{\rm{\eta }}}_{{\rm{E}}}=\frac{{{\rm{E}}}_{{\rm{d}}}\times 100}{{{\rm{E}}}_{{\rm{c}}}}$$

where I represent discharge current, Δt is the discharge time taken to complete the discharge cycle, m is the active mass of the electrode. η_E_ is energy efficiency, E_d_ and E_c_ represents the charge and discharges energy^31^.

The assembling of the symmetric device has been done using gel electrolyte (PVA/KOH), which works both as an electrolyte and a separator. To prepare the gel polymer electrolyte, 6 g of PVA was added to 6 M solution of KOH and then heating at 95 °C followed by firmly stirring for 2 h. To assemble a solid-state symmetric device, the gel electrolyte membrane was sandwiched between the two as-fabricated g-C_3_N_4_@ZnCo_2_O_4_ electrodes. The specific discharge capacity (*Q*_s_) of symmetric device calculated from the GCD curves according to Eq. 1, where m is the total mass of active materials on both electrodes of the assembled device. The energy density (*Ed*) and power density (*P*_*d*_) of the symmetric device is calculated using the following equations^32,33^.

3 $${E}_{{\rm{d}}}=\frac{I}{3.6\,m}\int Vdt$$

4 $${P}_{{\rm{d}}}=\frac{{E}_{{\rm{d}}}\times 3600}{\varDelta t}$$

where *I* describe the discharge current, *m* represents the mass of active material, the integral term is the area under the CD curve and Δt is the discharge time (seconds).

### Characterization techniques

The phase and structure of the samples were investigated by X-ray powder diffraction (XRD) diffractometer with radiation of Cu Kα (λ = 0.15405 nm). The surface morphology of samples was characterized by scanning electron microscope (SEM, JEOL JSM-6390LV, 20 kV). To study the elemental composition of synthesized samples X-ray energy dispersive analyzer was used. The Brunauer-Emmett- Teller (BET) measurements were used to calculate the specific surface area and pore size distributions, performed on a Belsorp Max system using nitrogen gas adsorption/desorption isotherms.

## Conclusions

We have developed a g-C_3_N_4_ supported ZnCo_2_O_4_ composite electrode using a simple hydrothermal method for improving the electrochemical performance of the electrode. The synergistic effect of g-C_3_N_4_ and ZnCo_2_O_4_ in g-C_3_N_4_@ZnCo_2_O_4_ hybrid composite shows a significant increase in a specific surface area along with a specific discharge capacity of 157 mAhg^−1^. Accordingly, g-C_3_N_4_@ZnCo_2_O_4_//gel electrolyte//g-C_3_N_4_@ZnCo_2_O_4_ symmetric supercapattery device is fabricated, which displays a high specific discharge capacity of 121 mAhg^−1^ at 4.6 Ag^−1^. The device exhibits the highest energy density of 39 Whkg^−1^ at a power density of 1478 Wkg^−1^. A good cycling stability performance with an energy efficiency of 75% is observed up to 10,000 cycles. This novel strategy establishes a stage towards the construction of a hybrid electrode with prominent performance, which is the present demand for energy storage devices.

## Supplementary information

Supplementary Information.
