# Supplementary material for: Designing of Carbon Nitride Supported ZnCo2O4 Hybrid Electrode for High-Performance Energy Storage Applications
Source: Sci Rep. 2020 Feb 6;10:2035. doi: 10.1038/s41598-020-58925-4 (PMC7005029; doi:10.1038/s41598-020-58925-4)
Supplement: Supplementary file 1 — Supplementary Information. [file 41598_2020_58925_MOESM1_ESM.docx]

**Designing of Carbon Nitride Supported ZnCo_2_O_4_ Hybrid Electrode for High-Performance Energy Storage Applications**

Meenu Sharma and Anurag Gaur^*^

Department of Physics, National Institute of Technology, Kurukshetra-136119, Haryana

**
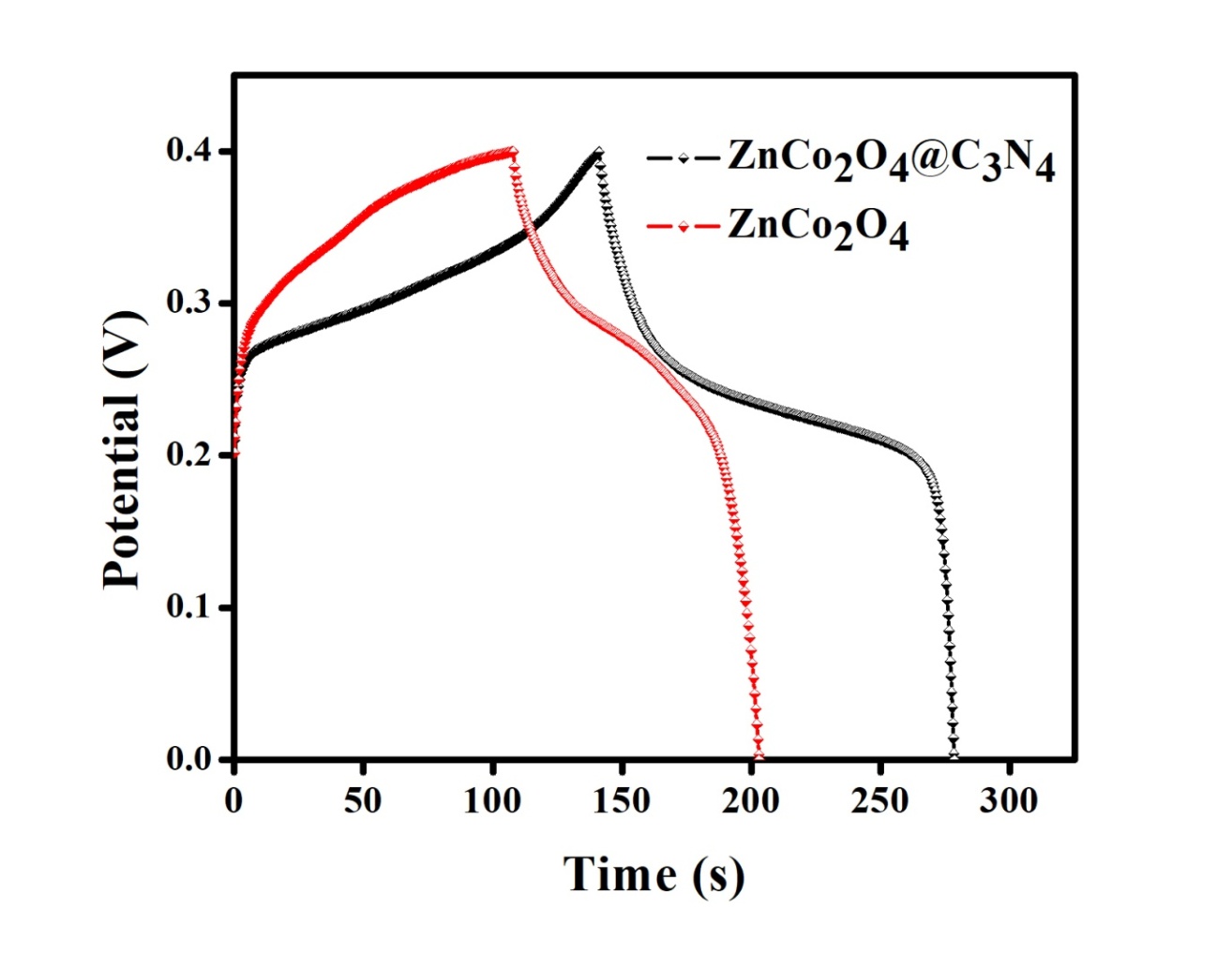
**

**Fig. S1:** GCD curves of ZnCo_2_O_4_ and g-C_3_N_4_@ZnCo_2_O_4_ electrodes at a constant specific current of 4 Ag^-1^.
